# Microfluidic-Based Droplets for Advanced Regenerative Medicine: Current Challenges and Future Trends

**DOI:** 10.3390/bios12010020

**Published:** 2021-12-31

**Authors:** Hojjatollah Nazari, Asieh Heirani-Tabasi, Sadegh Ghorbani, Hossein Eyni, Sajad Razavi Bazaz, Maryam Khayati, Fatemeh Gheidari, Keyvan Moradpour, Mousa Kehtari, Seyed Mohsen Ahmadi Tafti, Seyed Hossein Ahmadi Tafti, Majid Ebrahimi Warkiani

**Affiliations:** 1 School of Biomedical Engineering, University of Technology Sydney, Sydney, NSW 2007, Australia; hojjatollah.nazari@student.uts.edu.au (H.N.); sajad.razavibazaz@student.uts.edu.au (S.R.B.); 2 Research Center for Advanced Technologies in Cardiovascular Medicine, Tehran Heart Center Hospital, Tehran University of Medical Sciences, Tehran 14535, Iran; asieh.heirani@gmail.com (A.H.-T.); hosseinahmaditafti@yahoo.com (S.H.A.T.); 3 Department of Cell Therapy and Hematology, Faculty of Medical Sciences, Tarbiat Modares University, Tehran 14535, Iran; 4 Interdisciplinary Nanoscience Center (iNANO), Aarhus University, 8000 Aarhus, Denmark; sadeghqorbani@gmail.com; 5 Cellular and Molecular Research Center, School of Medicine, Iran University of Medical Sciences, Tehran 14535, Iran; h.eyni1990@gmail.com; 6 Department of Anatomical Sciences, School of Medicine, Iran University of Medical Sciences, Tehran 14535, Iran; 7 Department of Pharmaceutical Nanotechnology, School of Pharmacy, Zanjan University of Medical Sciences, Zanjan 45371, Iran; Khayati293@yahoo.com; 8 Department of Biotechnology, University of Tehran, Tehran 14535, Iran; fatemeh.gheidari@gmail.com; 9 Department of Chemical Engineering, Sharif University of Technology, Tehran 14535, Iran; k1moradpour@gmail.com; 10 Department of Biology, Faculty of Science, University of Tehran, Tehran 14535, Iran; Mousakehtari@gmail.com; 11 Colorectal Surgery Research Center, Imam Hospital Complex, Tehran University of Medical Sciences, Tehran 14535, Iran; smohsenahmadi1364@gmail.com; 12 Institute of Molecular Medicine, Sechenov University, 119991 Moscow, Russia

**Keywords:** droplet, microfluidics, regenerative medicine, stem cell therapy, tissue engineering

## Abstract

Microfluidics is a promising approach for the facile and large-scale fabrication of monodispersed droplets for various applications in biomedicine. This technology has demonstrated great potential to address the limitations of regenerative medicine. Microfluidics provides safe, accurate, reliable, and cost-effective methods for encapsulating different stem cells, gametes, biomaterials, biomolecules, reagents, genes, and nanoparticles inside picoliter-sized droplets or droplet-derived microgels for different applications. Moreover, microenvironments made using such droplets can mimic niches of stem cells for cell therapy purposes, simulate native extracellular matrix (ECM) for tissue engineering applications, and remove challenges in cell encapsulation and three-dimensional (3D) culture methods. The fabrication of droplets using microfluidics also provides controllable microenvironments for manipulating gametes, fertilization, and embryo cultures for reproductive medicine. This review focuses on the relevant studies, and the latest progress in applying droplets in stem cell therapy, tissue engineering, reproductive biology, and gene therapy are separately evaluated. In the end, we discuss the challenges ahead in the field of microfluidics-based droplets for advanced regenerative medicine.

## 1. Introduction

Regenerative medicine is an emerging therapeutic method to reconstruct the human body’s damaged organs and tissues [1]. This growing interdisciplinary science combines different approaches and technologies, such as medicine, stem cell biology, genetics, biomaterials, and chemistry. Regenerative medicine’s primary field is cell therapy that directly implements stem cells and their secretions—such as exosomes and extracellular vesicles (EVs)—for tissue repair [1,2]. The accompanying of stem cell technology with biomaterial sciences, nanotechnology, and manufacturing techniques has led to the advent of tissue engineering, trying to improve cells’ efficacy and functionality in injured tissues [3,4,5]. Moreover, the promising area of gene therapy resulting from tremendous improvements in genetics and molecular biology enables researchers to manipulate genes in cells [6]. The efficacy of all of these fields in regenerative medicine could not be improved without the provision of the stem cells being encapsulated safely in three-dimensional (3D) microenvironments mimicking their native home, or being confined in small rooms that prepare micro/nano-scale necessities and conditions for cellular manipulation and analysis [7,8]. Meeting this objective could significantly enhance the efficacy and success of stem-cell-based therapeutic methods, achievable by implementing droplets made by using microfluidic systems.

Droplet-based microfluidics is the technology of generating and manipulating small volumes of fluids (in the range of nL to pL) in immiscible phases. The droplets made using this technique can be produced at high frequency with desirable monodispersed sizes and well-defined volumes [9]. Moreover, well-controlled sequences of reproducible droplets can be obtained by implementing different geometries and patterns in microfluidic devices [10], which prepare desirable microenvironments for the isolation and encapsulation, culturing, gene editing, analysis, manipulation, and fusion of cells [11]. Therefore, they can provide a proper environment for cellular and molecular studies in different fields of regenerative medicine, including stem cell therapy, tissue engineering [12], gene therapy, and reproductive biology [13].

Microfluidic systems can provide safe and high-efficacy encapsulation methods for capturing stem cells inside aqueous droplets [14]. The droplets can be filled with specific culturing media for growing stem cells, or be filled with 3D hydrogels to mimic the native microenvironment of tissues for stem cells to proliferate and differentiate toward the desired adult cells [15]. Therefore, these droplets can mimic stem cells’ niches and improve complicated cellular complexes [16]. In addition to mimicking the stem cells’ niche, encapsulation of stem cells inside droplets can reveal their behavior and fates in different microenvironmental indications, such as cell–cell signals and possible cell–biomaterial interactions [17]. Moreover, droplets can simulate the biological and mechanical conditions inside niches of stem cells to aggregate and form mono/multicellular spheroids. Furthermore, the microenvironment created by droplets enables the development of intricate cellular clusters such as organoids via self-assembly and tissue formation. Droplets can also be appropriate platforms for performing high-throughput analysis and screening stem cells at a single-cell level to provide valuable information required for cell therapy and tissue engineering [17]. For instance, this technology enables us to study single stem cells’ gene expression profiles in different stages of differentiation. Moreover, the microenvironments made by droplets can prepare conditions necessary for other high-throughput analyses—such as RNA sequencing—more efficiently and cost-effectively [18].

Implementing microfluidics-based droplets with assisted reproductive technology (ART) techniques can also improve the efficacy of generating artificial uteri, stem-cell-derived gametes, and human cloning [19,20]. Furthermore, droplets can also prepare desired genetic manipulation conditions in gene-therapy-based therapeutic methods [21]. Droplets act as nano-scale platforms to improve the efficacy of correcting genetic disorders via the insertion of exogenous genetic materials into cells based on non-viral vector systems. Therefore, the side effects caused by viral infections during gene editing of cells can be reduced by encapsulating cells and desired genomic materials inside droplets and implementing physical forces such as electric, optic, and hydrodynamic forces.

**Figure 1:**
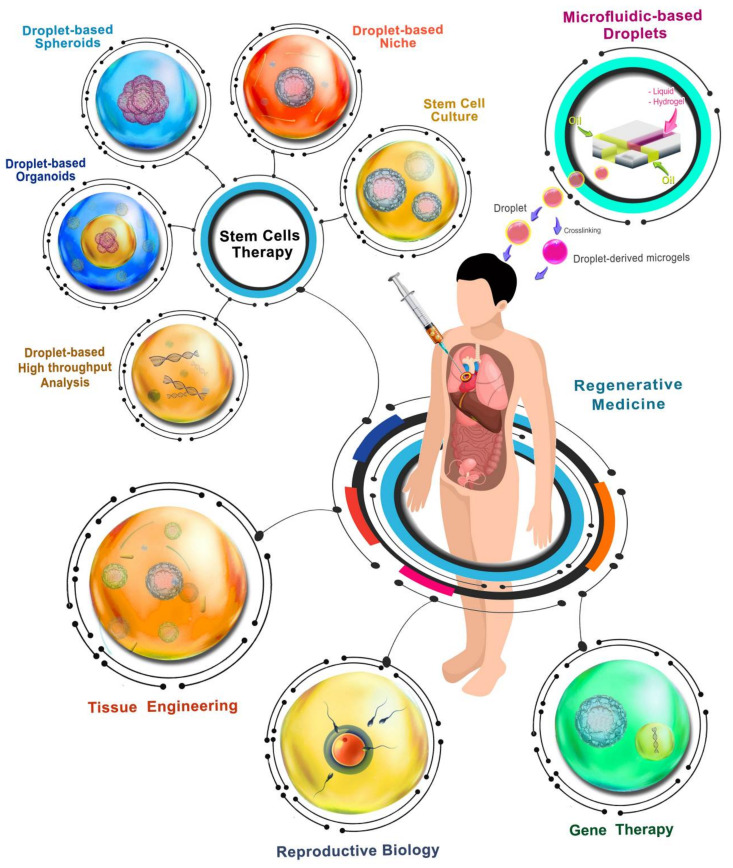
Microfluidics-based droplets for advanced regenerative medicine: Their applications in stem cell research (cell culture, niche engineering, spheroid forming, organoid generation, and high-throughput analysis), tissue engineering, reproductive biology, and gene therapy.

In this review, we present the state-of-the-art microfluidics-based droplet technologies for preparing microenvironments needed for the main domains of regenerative medicine, including stem cell therapy, tissue engineering, reproductive biology, and gene therapy. This review is organized into microfluidics-based droplet generation methods and the latest applications of droplets in each regenerative medicine domain (Figure 1). Finally, our conclusion and future remarks for implementing microfluidics-based droplets to bridge the gaps between the regenerative medicine lab and clinical practice are discussed.

## 2. Microfluidics-Based Droplets

Microfluidics technology has undergone outstanding progress after its debut in the 1990s, and has shown massive functionality for biomedical applications [22,23,24]. Furthermore, the capacity of microfluidics to manipulate multiphase systems and generate monodispersions of polymer particles, emulsions, bubbles, and droplets has increased due to vast advances in microfabrication over the past decade [25]. Microfluidics-based systems implement the fundamentals of two-phase dynamics in microchannels for fabricating droplets for regenerative medicine purposes. These platforms facilitate droplet fabrication based on emulsification methods within their microchannels, and generate monodispersed droplets. Moreover, these systems can eliminate some common challenges in droplet generation using bulk techniques [26]. One of the biggest challenges in non-microfluidic methods is mixing two fluids in the bulk volume to enhance the turbulence, causing the break-up of droplets due to shear flows. Microfluidic devices can precisely control individual droplets, reduce sample consumption, and decrease polydispersity [27]. Furthermore, the unique convective flow profile within individual droplets enhances the mixing, leading to significant improvement of heat and mass transfer and acceleration of reactions [28]. For the aim of droplet generation, a variety of geometries and patterns can be implemented. These systems can also be integrated with automation systems and computers for versatile manipulation of the droplet formation process.

Droplet formation and manipulation in microfluidic devices can be either passive or active [29]. Passive droplet formations include co-flow (coaxial), crossflow (mainly in T-junction), and flow focusing [30]. In co-flow systems, samples are introduced via coaxial microchannels. The inner channel is assigned for the dispersed fluid phase, while the outer channel is responsible for the continuous phase. For the crossflow designs, the continuous and the dispersed fluid meet at an angle, where the most common case is a T-junction channel at an angle of 90 degrees. In the flow-focusing category, the continuous and the dispersed flow streams are introduced coaxially inside a confined region, which leads to the generation of droplets. The interfacial tension, viscosity, and flow rate ratio of continuous and dispersed flows define the gap, size, and generation rate of droplets. Active droplet formation and manipulation also involves electric, magnetic, and/or acoustic forces. Within the electric control, both alternating and direct current can be used (see the EWOD concept in Section 5). The droplet size can be precisely controlled by adjusting the electric field strength in the electrical methods. In dielectrophoresis (DEP), through the use of an electric field, uniform droplets can be generated by pulling the droplets from the reservoir of fluid. For the magnetic fields, the magnetic control occurs via ferrofluids, which are magnetic nanoparticle suspensions in an aqueous or oil-based carrier [31]. In droplet microfluidics, ferrofluids can be considered the dispersed- or continuous-phase flow. Droplets in magnetic-assisted droplet microfluidics can be characterized using the magnetic bond number, which is defined as the ratio of the magnetic force to the interfacial tension strength. Acoustic forces can also be helpful in droplet generation. For instance, surface acoustic wave devices can be utilized to induce mechanical vibration, assisting in droplet generation [32].

Many factors affect droplet formation. Therefore, proper comprehension of droplet formation and its dynamics is enlightening for utilizing its applications [33]. In passive droplet microfluidics, immiscible flow phases exist, generating both individual volumes of fluids and moving interfaces. Within each individual droplet, the convective flow profile eases the mixing. The linear Stokes equations govern the microfluidic droplet dynamics [34]. However, certain nonlinearities exist because of the two-phase flow interface and the variable interfacial tension. In passive microfluidics and pressure-driven flow, the interface deformation and the associated droplet breakup are affected by the channel junction geometry design and the local fluid flow. In droplet microfluidics, dimensionless numbers characterize the fluid behavior. The first crucial dimensionless number is the Reynolds number, defining the ratio of inertial to viscous forces. However, the Reynolds number is usually small; therefore, inertial forces become negligible. Another substantial dimensionless number is the capillary number, Ca, which identifies the ratio of viscous strength to interfacial forces. Indeed, two competing effects of the interface extension and deformation, which are mainly caused by deformation resistance and shear stress, affect the droplet formation. The other important dimensionless number is the Weber number, which defines the ratio of inertial to interfacial tension. At high flow rates, the transition of individual droplets to continuous jets is affected by inertial effects. Although the dimensionless numbers mentioned above are the most critical numbers in droplet microfluidics, other factors such as buoyancy, gravity, or elastic effects also exist under certain circumstances, and must be evaluated through the definition of proper dimensionless numbers [33].

**Figure 2:**
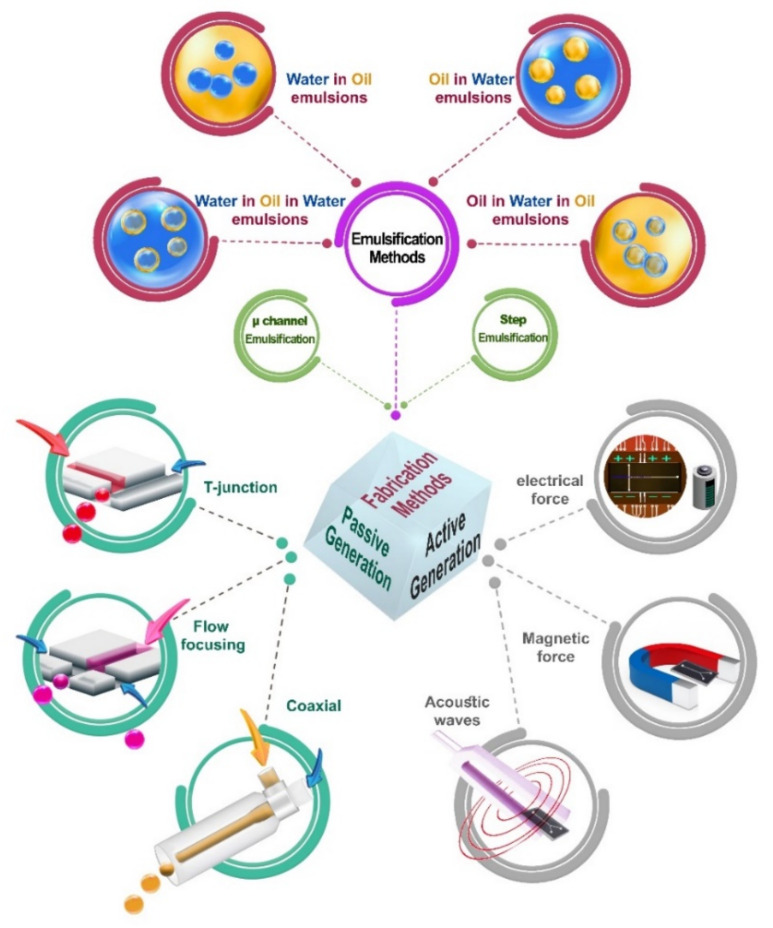
Microfluidics-based methods for droplet generation. The emulsification methods, types of droplets, geometries used in the passive generation, and forces can be implemented for the active generation of droplets.

The emulsions used for droplet generation inside microfluidic devices are classified into four system structures: oil-in-water, water-in-oil, water-in-oil-in-water, and oil-in-water-in-oil emulsions. The oil-in-water emulsions are systems consisting of oil droplets dispersed in an aqueous phase; these emulsions have been used to encapsulate and deliver various bioactive lipids, fatty acids in food products, and oil-soluble drugs for pharmaceutics [35,36]. The second category is water-in-oil emulsions—systems of water droplets dispersed in an oil phase; these emulsions have been utilized in several industries, including foods, cosmetics, chemical synthesis, and printers. These suspended aqueous droplets can encapsulate water-soluble ingredients (such as polymers and hydrogels) or living cells (including stem cells and progenitor cells) after crosslinking, and are highly useful in the fields of tissue engineering and regenerative medicine [37]. There are also water-in-oil-in-water and oil-in-water-in-oil emulsions, which are known as multiple emulsions consisting of water and oil droplets dispersed within larger opposite-phase droplets, e.g., the water-in-oil-in-water emulsions are double emulsions in which oil droplets enclosing water droplets are dispersed in water (Figure 2). These multiple emulsions are widely used in the food industry [38], cosmetic production, pharmaceutical research, and chemical separation [39,40]. Furthermore, many methods exist for droplet manipulation.

## 3. Microfluidics-Based Droplets for Stem Cell Therapy

Stem-cell-based therapy is a regenerative medicine division in which stem cells are implemented for repairing tissues and organs [41]. Due to the tremendous advances in stem cell technologies over the past decade, this therapeutic approach has extended to the clinical trial stages to treat various diseases [42]. The advent of different types of stem cells—including pluripotent stem cells (PSCs), mesenchymal stem cells (MSCs), embryonic stem cells (ESCs), and induced pluripotent stem cells (iPSCs)—has made advanced stem cell therapy a game-changer in regenerative medicine [43]. The setup of precise derivation methods and treatment of cells with specific culturing media is necessary in order to differentiate these stem cells into the desired functional adult cells. Such environmental conditions can be created inside droplets made via microfluidic technology. In this section, the utilization of droplets for culturing and differentiation of stem cells, simulating stem cell niches’ microenvironments, and the formation of spheroids and organoids is reviewed. In the end, the droplet-assisted methods for isolation and single-cell analysis of stem cells are described.

### 3.1. Droplet-Based Stem Cell Culture

The technologies associated with the expansion, editing, and transplantation of stem cells and progenitor cells play an essential role in cell therapy and regenerative medicine [44]. Three significant characteristics identify stem cells: first, they have self-renewal capacity; second, they are unspecialized cells; third, they can differentiate into specialized subpopulations [45]. These cells reside in specific dynamic 3D microenvironments in the body called niches, which provide complicated biochemical and biophysical cues that aid in these cells’ survival and determine their fate [46]. The advancement of microfluidics-based droplets can enhance our perception of the regulating functions involved in microenvironmental signaling, significantly improving cell therapy’s chance of success. The generation of monodispersed micro-scale droplets via microfluidic systems provides stem cell researchers with facilities to encapsulate stem cells inside 3D microgels with the desirable microenvironment, removing some challenges in stem cell therapy. For instance, the low retention and homing of transplanted stem cells after transplantation dramatically reduce their therapeutic effects on patients. In other words, it is necessary to support transplanted cells’ retention and chemotaxis in order to achieve long-term and efficient cell therapy [47,48]. Therefore, we need approaches such as droplet carriers or droplet-derived microgels that prolong cell culture time before and after transplantation. Another challenge in regenerating damaged tissue is the simulation of the 3D structure of tissue, which provides a range of physiologically more significant features than 2D, such as culturing stem cells or sphere formation to form multicellular tissue cultures in vitro for further implantation [49].

Droplets can enhance the efficacy of one of the primary stem cell sources in cell therapy—MSCs [50]. These cells can be separated from the bone marrow, adipose tissue, amniotic fluid, amniotic membrane, umbilical cord, and placenta, which secrete different anti-inflammatory, angiogenic, and anti-fibrotic factors, and cause immunomodulation without noticeable activation of any immune responses [51]. The low homing capabilities in targeting of tissues by MSCs can be addressed by encapsulating MSCs inside 3D droplet-derived hydrogels [52,53]. Moreover, these elements are suitable for the simultaneous carrying of sensitive biological cargoes, including nucleic acids, proteins, drugs, nanoparticles, and living cells [54,55]. By controlling the droplet-derived microgels’ porosity, the presence of immunostimulatory agents produced by allogeneic or xenogeneic cells transplanted to the immune system in surrounding tissues and capillaries could be reduced. At the same time, the diffusion of oxygen and cellular waste products is preserved. Microgel porosity can be easily controlled by different hydrogel biomaterials and concentrations [56]. Many studies have investigated the impact of encapsulating MSCs on the secretion of biochemical agents and signaling proteins made by cells inside hydrogels. For instance, Headen et al. used a two-layer parallel microfluidic system to encapsulate human MSCs in synthetic microspheres with less than 100 μm diameter; they observed that these 3D microspheres increased cells’ viability and enhanced the secretion of vascular endothelial growth factor (VEGF), which is necessary for angiogenesis [57]. VEGF is an exclusive mitogen for vascular endothelial cells, which induces proliferation, promotes migration, and inhibits apoptosis of endothelial cells [58]. In addition to isolating transplanted cells from the host immune system, 3D gels allow engineering of the cell-laden microenvironment, which presents cells with the stiffness of matrix–adhesive ligand interactions. These factors significantly influence the secretory function and differentiation of encapsulated stem cells [59,60].

**Figure 3:**
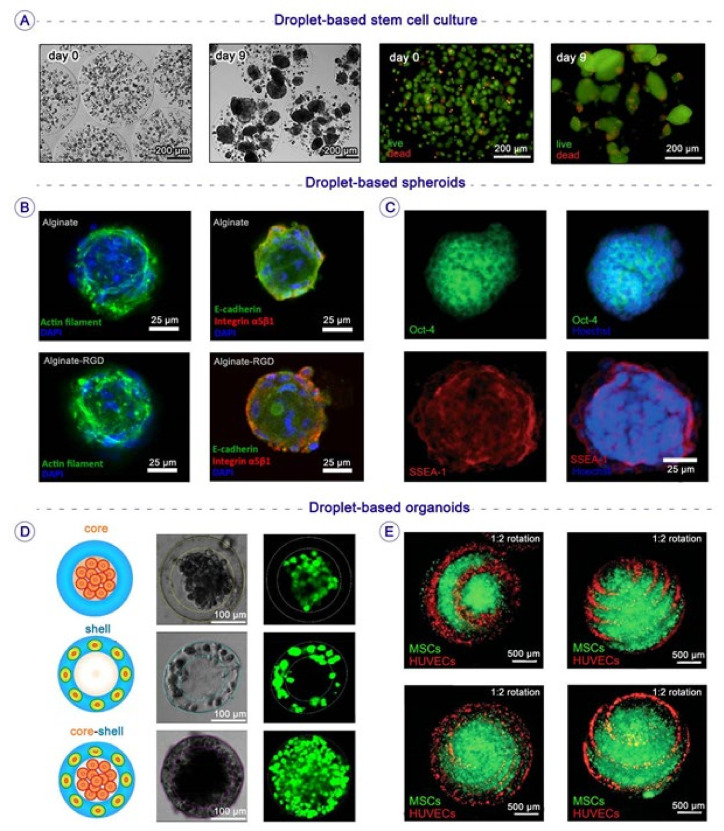
Microfluidics-based droplets for generating 3D culture conditions, niches, spheroids, and organoids for stem cell therapy applications: (**A**) Viability and morphology of encapsulated hESCs at different timepoints after encapsulation in alginate microcapsules (viable cells: green GFP; non-viable cells: red PI) [63]; Copyright, Elsevier. (**B**) Immunostaining for E-cadherin and integrin for spheroids encapsulated in alginate or alginate/RGD microgel after three days [69]; Copyright, Nature. (**C**) Immunohistochemical staining of pluripotency marker proteins (Oct-4, green; SSEA-1, red; Hoechst, blue) in the embryonic spheroids [70]; Copyright, The Royal Society of Chemistry. (**D**) Spatial assembly of different cells in the 3D core–shell scaffolds. Up: HepG2 cells are confined in the core by the hydrogel shell. Middle: NIH-3T3fibroblasts immobilized by the crosslinked alginate network in the shell. Down: Simultaneous assembly of hepatocytes in the core and fibroblasts in the shell, forming an artificial liver in a drop [73]; Copyright, The Royal Society of Chemistry. (**E**) Construction of organoids through the co-culture of HUVECs and hMSCs in spiral-based microspheroids. HUVECs were spirally distributed on the surface of the spheroids, with a composition ratio (QHUVECs:QhMSCs) of 1:2 [74]; Copyright, Wiley Online Library.

Human embryonic stem cells (hESCs) are also popular stem cell sources in clinical trials; they can be endlessly expanded in culture and differentiated into any given cell type in the body [61]. The differentiation of hESCs is generally performed either in 3D aggregates known as embryoid bodies (EBs), or in two 2D monolayer cultures. Droplets can also assist in the encapsulation of hESCs inside biomaterials and microenvironments [62]. For instance, Chayosumrit et al. established a 3D model to expand and differentiate hESCs and encapsulate them in calcium alginate microcapsules. The microgels induced cellular interactions, which are essential for preserving both pluripotency and differentiation of hESCs, allowing separation of hESCs from fibroblasts and providing immune isolation during transplantation (Figure 3A) [63].

### 3.2. Droplet-Based Spheroids

When stem cells settle in their native 3D niche microenvironment, they assemble with other cells and form aggregates, called cell spheroids [51]. These stem cells inside spheroids can prepare complicated interactions with other types of stem cells, progenitor cells, stromal cells (via their secretary molecules), and extracellular matrix (ECM) molecules, leading to their differentiation, activation, and performance. The in vitro encapsulation and culturing of stem cells inside droplets with similar physiological and mechanical properties to the native niche can enable us to address such challenges [64].

Despite developing a variety of techniques for in vitro spheroid formation—including hanging drop, spinner flask, and suspension culture—over the past decades, some limitations decrease the efficacy of spheroids for research and clinical purposes, including the laborious procedure, and the limited diffusion of oxygen and nutrients to the spheroids [65,66,67]. The microfluidics-based droplets can act as platforms for high-throughput culturing and precise size control of spheroids inside 3D microenvironments to address these issues. Furthermore, the fabrication of stem cell multicellular spheroids with controlled dimensions, which are in high demand in regenerative medicine, is achievable using droplets because they can provide different cells inside spheroids with the ability to interact, regulating biological processes such as hemostasis and disease development. In vitro formation of multicellular spheroids enables the intercellular interactions required for cell viability, phenotyping, and function maintenance of stem cells [68].

In terms of multicellular spheroids, Chan et al. developed a method for generating double-emulsion water-in-oil-in-water (*w*/*o*/*w*) droplets for high-throughput production of spheroids via droplets encapsulating different cell types, such as human mesenchymal stem cells (hMSCs); they applied these droplets as bioreactors to accelerate the growth and formation rates in spheroids; moreover, they extracted these spheroids and encapsulated them in alginate and alginate/RGD (arginine-glycine-aspartic acid) 3D microenvironments, and found that the incorporation of modified alginate enhanced the osteogenic differentiation of spheroids (Figure 3B) [69]. The liquid microenvironment of droplets can also create cellular spheroids via their cell–cell interactions in a pre-hatching embryo. Agarwal et al. constructed a core–shell microcapsule to imitate the 3D structure of pre-hatching embryos with a hydrogel alginate shell and an aqueous core of embryonic cells; their results demonstrated a survival rate of over 92% in encapsulated embryonic cells. Inside these microfluidic-derived droplets, single cells could turn into embryonic bodies (EBs) consisting of almost 20 cells, while hundreds of cells are required to form embryonic bodies via the hanging drop method. Agarwal et al. also observed a significant increase in pluripotency gene expression compared to in 3D culture (Figure 3C). Finally, they differentiated these EBs into beating cardiomyocytes with the aid of only a small molecule instead of a complex combination of growth factors [70].

One of the most crucial requirements to vastly employ spheroids in regenerative medicine, drug discovery, and pharmaceutics is a production process that is cost-effective, easy to implement, and not prone to human error. A variety of research groups are attempting to advance robotized droplet microfluidic platforms to produce practical microspheres for cell encapsulation and culture on a large scale. For instance, Langer and Joensson developed an automated microfluidics-based droplet generation system for spheroid formation; they fabricated scaffold-free cell spheroids with highly monodisperse droplets, at a high production rate of 85,000 spheroids per microfluidic device per hour; furthermore, their platform consisted of main steps, including droplet generation, formation, and recovery, as well as dispensing of spheroids [71]. In another study, Cedillo-Alcantar et al. developed an automated microfluidic system that produced droplets to cultivate as well as biosensing hepatic spheroids. The droplets were able to mimic hormones’ physiological microenvironment in the hepatic portal circulation and support the long-term preservation of primary hepatocytes. Additionally, spheroids’ biochemical responses inside droplets (secretion of glucose and albumin) were monitored in a real-time manner using an enzymatic assay [72].

### 3.3. Droplet-Based Organoids

Organoids are intricate cell clusters made of different stem/progenitor cells of specific organs that form complex tissue-specific structures in the presence of 3D ECM microenvironments via self-assembly. Improving the in vitro 3D culture systems to mimic the native microenvironment of organoids in the body and generate organoids that can produce vessel-like structures for fluid transportation is a demanding concern that can be addressed using droplet-derived microgels. Moreover, the challenge of deficient nutritional supply in organoids can cause limitations of their size, survival, and functionality, which can be addressed via a microfluidics-based droplet-encapsulating approach [75].

**Figure 4:**
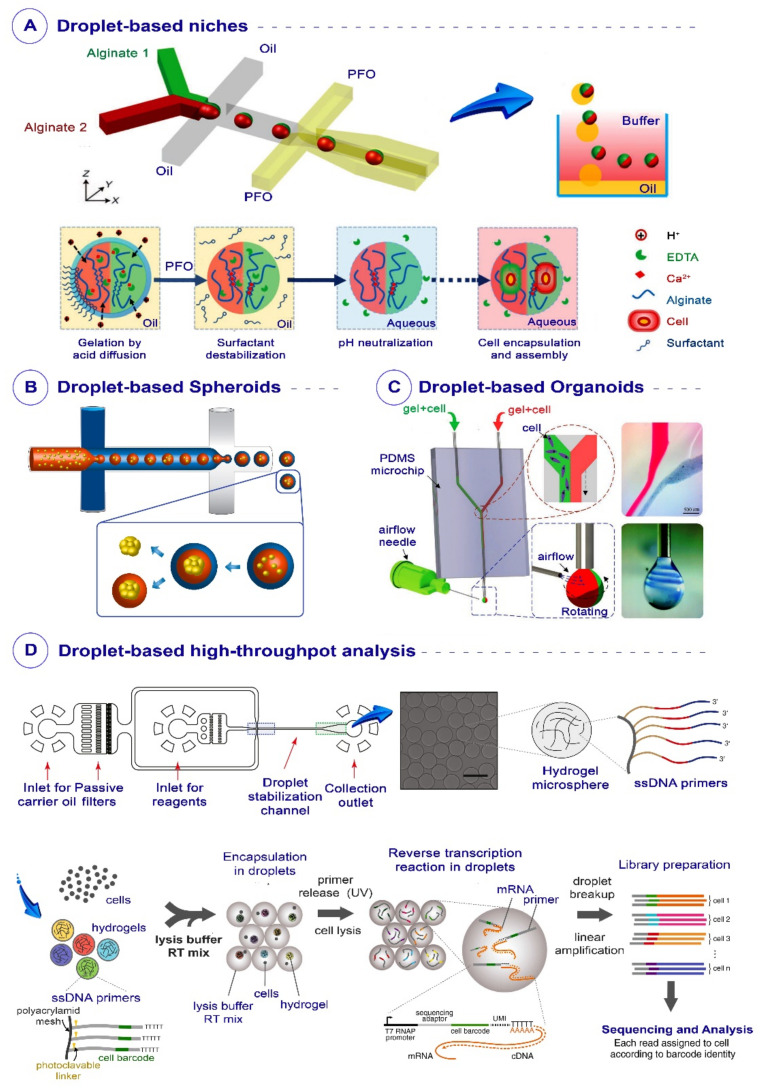
Microfluidics-based methods for droplet fabrication for stem cell therapies: (**A**) Up: Design of a microfluidic device to produce single emulsion drops consisting of multiple aqueous phases; the alginate droplets are solidified inside the chip and are immediately collected inside cell media. Down: The detailed mechanism to form multi-compartment alginate microgels [69]; Copyright, Wiley Online Library. (**B**) Rapid formation of multicellular spheroids after assembly of encapsulated cells. The spheroids can be released with or without microgel encapsulation [76]; Copyright, Nature. (**C**) Design of an airflow-assisted 3D bioprinter with a microfluidic nozzle to manufacture 3D spiral-based cell-laden spheroids. Sodium alginate solutions are extruded from the microchip and rotated by the airflow [74]; Copyright, Wiley Online Library. (**D**) Design of a droplet-based method for single-cell sequencing. Hydrogel microspheres are made containing barcoded oligo(dT) primers and sequencing adaptors. Each barcoded hydrogel microsphere is encapsulated with a single cell into a droplet with lysis buffer and reverse-transcription mix. Primers are released regarding UV exposure, and cDNA synthesis is performed in each droplet. Droplets are then broken, and barcoded cDNA of each cell is linearly amplified, followed by sequencing [76]; Copyright, Elsevier.

**Figure 5:**
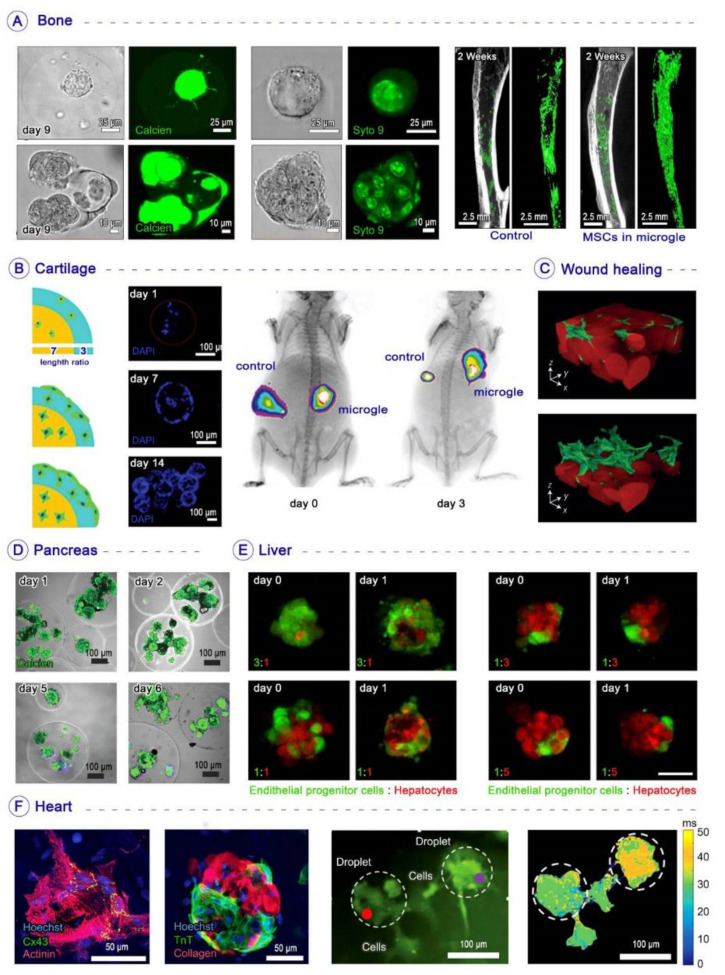
Microfluidics-based droplets for the fabrication of microgels in tissue engineering applications: (**A**) Left: Confocal microscopic images showing MSCs encapsulated in RGD-alginate microgels cultured in vitro for bone regeneration. Due to alginate microgels’ stiffness, cells remained spherical even though they proliferated within the matrix. Middle: The confocal microscopic images of MSCs stained by SYTO 9 nuclei staining of the microgels. Right: 2D and 3D reconstructed micro-CT images of a tibia’s medullary cavity after two weeks of in vivo transplantation [100]; Copyright, Elsevier. (**B**) Left: In vitro biological characterization of self-assembled BMSC-laden gelatin–hyaluronic acid microgels after 14 days. The gelatin–hyaluronic acid microgels were divided into core and shell parts with length ratios of 7:3. (DAPI-stained cell nuclei). Right: BMSC-laden microgels were hypodermically implanted into the right of the nude mouse, and pure BMSCs were hypodermically implanted into the left of the nude mouse as a control [103]; Copyright, Wiley Online Library. (**C**) Accelerated wound healing by fabrication of injectable microporous gel scaffolds assembled from annealed building blocks [108]; Copyright, Nature. (**D**) Microfluidics-based PEG-4MAL microgels retain the viability and function of encapsulated human islets after microencapsulation [111]; Copyright, Wiley Online Library. (**E**) Microfluidics-based production of microencapsulated functional hepatocyte spheroids with different cell portions [115]; Copyright, Wiley Online Library. (**F**) The morphology and contractility of the encapsulated cardiac cells. Left: Immunostaining of α-sarcomeric actinin (red), connexin-43 (green), and nuclei (blue). Immunostaining of collagen (red), troponin T (green), and nuclei (blue). Middle: In vitro migration and function of encapsulated cardiac cells. Right: Calcium imaging between two adjacent droplets [118]; Copyright, Wiley Online Library.

Safe encapsulation of different types of cells in liquid microenvironments or 3D microscale constructions made of biocompatible ECM is a great challenge in the development of functional organoid models, achievable using droplet-based microfluidics [76] (Figure 4A). For instance, the liver comprises various cell types, including primary hepatocytes, hepatic stellate cells, Kupffer cells, endothelial cells, and fibroblasts. The proper liver-specific functions are achievable when these cells are cultured together in 3D cell co-culture systems. Chen et al. utilized a flow-focusing microfluidic device to produce droplets containing an aqueous core and an alginate hydrogel shell. To generate the human liver model in each droplet, they encapsulated hepatocytes and fibroblasts in the core and hydrogel shells, respectively. The co-culture of hepatocytes and fibroblasts increased homotypic and heterotypic cell–cell interactions, followed by high liver-specific functions inside droplets. Additionally, the alginate shell’s high permeability resulted in the high cell viability of micro artificial organs after more than 10 days (Figure 4D) [73]. In another study to produce heterogeneous human organoids, Zhao et al. utilized a microfluidic nozzle to improve the efficacy of an airflow-assisted 3D bioprinter for the printing of cell-laden spiral microarchitectures; they encapsulated human umbilical vein endothelial cells (HUVECs) and hMSCs inside spiral-based microspheroids to establish a complicated co-culture 3D microenvironment for osteogenesis and angiogenesis in these organoids [74]. These cell-laden spheres supported in vitro production of osteogenic nodules in functional organoids (Figure 4E and Figure 5C) [74].

### 3.4. High-Throughput Analysis of Stem Cells Using Droplet-Based Techniques

The high-throughput analysis of each stem cell in a heterogeneous population can provide valuable information about their biological systems’ complex behavior during clinical applications. The confining of single cells inside droplets can enhance the efficacy of laborious, expensive, and low-throughput molecular techniques such as Sanger sequencing, real-time PCR, blotting techniques, and fluorescent microscopy. Moreover, the implementation of droplets in available high-throughput cell analysis techniques—such as polymerase chain reaction [77], DNA/RNA sequencing [78], enzyme kinetics [79] and assays [80], protein crystallization [81,82], protein expression quantification [83], protein quantification mass spectrometry (LC–MS), and GC–MS metabolomics—makes it more important to be able to analyze single cells. Therefore, the high potency of droplet-based microfluidic techniques makes them excellent platforms for single-cell analysis and small samples, such as stem cells, tumors, and micro-biopsies [84]. For instance, Klein et al. designed a method for barcoding, capturing, and profiling transcriptomes, with quick collection, low technical noise, and no limitations regarding the number of cells; they encapsulated single ESCs, which are highly heterogeneous due to their pluripotency inside droplets, and then performed all of the RNA sequencing steps on an individual basis [85].

The accurate isolation and analysis of distinct cells can provide critical information on various biological parameters and processes in stem cell research—especially gene- and cell-based therapies. Conventional methods of cell separation to date—including fluorescence-activated cell sorting (FACS), magnetic-activated cell sorting (MACS), density centrifugation, limiting dilution, and immunoaffinity—are not cost-effective, need a large amount of input sample, have low throughput, and are limited to specific applications [86]. The integration of microfluidic systems producing droplets can improve cell sorting and analysis via the above techniques. Furthermore, these picoliter-sized droplets provide an isolated environment for the distinct reactions of many biological and chemical assays.

The expression of both intracellular and extracellular proteins is among the critical characteristics of stem cells that can be screened or quantified at the single-cell level using droplets. The proteins of stem cells should be analyzed, because they play a crucial role in stem cell viability, metabolism, differentiation stages, generation of recombinant proteins or antibodies by recombinant or hybridoma cells, and finding protein-producer colonies for immunological and pharmacological purposes. The routine techniques for this aim—including fluorophore–antibody detection of proteins via flow cytometry, immunohistochemistry, Western blotting, immunocytochemistry, and enzyme-linked immunosorbent assay (ELISA)—are expensive, time-consuming, and need a large number of cell samples and reagents; moreover, they cannot enable us to screen rare cell phenotypes that may be of high importance as colonies of the expensive drug–protein producer, or as specifically characterized and efficient stem cells or progenitor cells. Microfluidics-based droplets make the screening and detection of rare cell phenotypes at a single-cell level possible. The small volumes of liquids inside the cell-laden droplets increase the concentrations of secreted biomolecules such as monoclonal antibodies to levels that are easily detectable using diagnostic methods. For instance, Koster et al. designed a droplet-producing microfluidic system that could detect a low amount of released antibodies secreted from single hybridoma cells just 6 h after secretion using the kinetic ELISA method [87].

Next-generation sequencing (NGS), by providing the possibility of whole-transcriptome sequencing at a single-cell level, has emerged as a novel technology in the field of stem cells; it reveals the heterogeneity of gene expression profiles in cell populations related to different stages of cellular differentiation or function. Whole-exome sequencing gives us a wide range of data on gene expression profiles, compared to high-throughput DNA/RNA arrays that are not whole-genome-wide. Microfluidics has helped scientists to perform next-generation sequencing at the single-cell level in order to help analyze heterogeneous cell populations and stem cells. For instance, Streets et al. developed a microfluidic device to capture and lyse single cells and synthesize cDNA from mRNAs via a next-generation sequencing platform. Using their device, they prepared 94 libraries from single mouse ECSs, and improved RNA detection sensitivity made it possible to reconstruct a majority of the bulk transcriptome from 10 single cells with 0.2 M reads per cell. Enhanced measurement precision and technical throughput helped to distinguish variations in expression between and within different types of murine embryonic cells [88]. Moreover, microfluidics-based droplets have provided scientists with numerous achievements in single-cell analysis. For instance, Klein et al. reported developing a high-throughput system that can encapsulate single cells and lysis buffer, reverse-transcription reaction mix, and hydrogels containing barcoded sequencing primers into droplets. After encapsulation and lysis of cells, ultraviolet (UV) treatment of droplets releases barcoded primers from hydrogels for cDNA synthesis. Following linear amplification and breakup of droplets, next-generation sequencing (NGS) makes specific reads from each cell’s genetic content regarding sequencing adaptors and unique barcodes on the cDNA. Therefore, Klein et al. applied this approach to murine ESCs after withdrawal of leukemia inhibitory factor (LIF), showing the heterogeneous onset of differentiation in this cell population and revealing gene expression relationships; their method showed acceptable reproducibility of data and a low noise baseline (Figure 4D) [85].

Zilionis et al. established a droplet microfluidics platform called inDrops for whole transcriptomics or genomics analysis of more than 15,000 individual cells in one hour, with minimal reagent use. More than 75% of cells in a sample are encapsulated into nanoliter aqueous droplets with hydrogel bead cores that bear barcoding primers. Following lysis in droplets, barcoded cDNA of cells is synthesized from mRNA via barcoding primers by reverse transcription, and then an RNA-Seq library is prepared. Zilionis et al. have used inDrops for murine primary immune cells and murine ESCs, and showed that this is the best method of single-cell transcriptomics for limiting samples consisting of less than 200,000 cells, such as rare stem cell populations or tumor biopsies [89]. Another microfluidics-based droplet technique called Drop-Seq, which Macosko et al. implemented for transcriptome analysis of murine retinal cells, had a lower rate of 2–4% of cells barcoded [90]. Applying such a high-throughput genome-wide expression profiling technique in a single cell enables scientists to precisely search for gene expression variations during stem cell culture and differentiation.

## 4. Microfluidics-Based Droplets for Tissue Engineering

Tissue engineering is defined as an interdisciplinary field—a combination of engineering, nanotechnology, biomaterials, and stem cell science to develop functional tissue-like structures for the replacement, repair, and rehabilitation of damaged tissues/organs [91]. During the past decade, various scaffolds with different shapes, morphologies, and mechanical properties have been developed using different materials for engineering a variety of tissues, among which cell-laden scaffolds were more successful in mimicking the 3D environments of desired tissues. These types of scaffolds should be highly biocompatible, with controlled degradability, mechanical stability, uniform size, and suitable mass transport functions [92].

To date, numerous materials have been applied for encapsulating cells in the cell-laden scaffold, among which hydrogels were more successful in mimicking the native microenvironment of human tissues for regulating stem cell proliferation and differentiation, synthesis of ECM proteins, and cell–matrix and cell–cell biochemical and mechanical interactions. Cell-laden hydrogels are usually produced by incorporating cells in precursor solution, followed by gelation using bulk- or microfabrication techniques. The encapsulation of cells within macroscopic hydrogels leads to low survival, incomplete cell–cell and cell–matrix interactions, and poor nutrient exchange because of the low diffusion rate in the scaffold [93]. One of the difficulties in the macro-scale 3D culture systems is that they are not suitable for long-term cell culture, because the maximum oxygen diffusion inside scaffolds is usually limited to 400 μm. On the other hand, the size of scaffolds made by routine methods is much bigger than this limit, and most of the encapsulated cells lose their functionality and viability in a short time. The cells encapsulated in microgels with a diameter of 100–400 μm can survive and proliferate safely. The number of cells encapsulated in the microgel and total cell encapsulation efficiency can be controlled via microfluidic techniques. In contrast, cell-laden microgels produced via microfabrication methods have a large surface-area-to-volume ratio, and can improve the efficient mass transfer and different nano-scale interactions between cells and their surrounding ECM [54]. These microgels can assemble inside damaged parts after injection and form engineered tissue-like constructs with similar morphology and functions to native tissue. The cell-laden microparticles can be manufactured through numerous techniques, one of which is droplet microfluidics.

Droplets made by microfluidic systems can prepare aqueous microenvironments to encapsulate cells and monomers of hydrogel solutions to be polymerized and form cell-laden microgels. These spherical cell-laden 3D scaffolds mimic the microenvironments needed for the proliferation and differentiation of stem cells, and generate the desirable mechanical, chemical, and physiological properties required for supporting damaged organs [94]. These systems can also encapsulate different types of cells, as well as biomolecules such as growth hormones, exosomes, biological stimulators, and various nanoparticles [95]. Therefore, the fabricated microgels can ultimately serve as functional building blocks for injection to the tissue constructs. In addition to the cell-encapsulating capacity of microfluidic systems, cells that can be cultured on the surface of the microfluidic microgels have several advantages: First, cells grown on the surface are not restricted, and can proliferate quickly within a short time. Second, detachment of cells culturing on the microgel surface is relatively easy without enzymatic operations, via mechanical separation methods or thermally mediated degradation. In the following section, we will briefly overview the applications of microparticles made using droplet technology in bone, cartilage, skin, pancreas, liver, and cardiovascular tissue engineering.

### 4.1. Bone Tissue Engineering

The employment of advanced technologies such as tissue engineering and stem cell therapy for advancing bone regeneration is highly demanding [96]. Despite the vast progress in bone tissue engineering, researchers and clinicians face serious challenges, such as low retention and low survival and transplanted stem cells’ metabolic activity in the host body. Microfluidics-derived droplets can help to prepare acceptable microenvironments enriched with biological cues of native bone tissue, and enhance the survival, proliferation, and osteogenic differentiation of stem cells. For instance, Moshaverinia et al. developed injectable hydrogel microspheres made of RGD (as a biological cue) and alginate for the encapsulation of periodontal ligament stem cells (PDLSCs) and gingival mesenchymal stem cells (GMSCs); their results showed that adding RGD to microspheres’ structure improved the viability and osteogenic differentiation of encapsulated cells and the expression of osteogenic markers such as RUNX family transcription factor 2 (Runx2), alkaline phosphatase (ALP), and osteocalcin in both in vitro and in vitro conditions. These cell-laden microspheres, which were less than 500 µm in diameter, were half the size of the ones obtained via bulk methods. This approach improved the transport of nutrients, oxygen, and waste products through the microspheres, resulting in high cell viability [97].

In addition to implementing biochemical cues in spheres, the rapid generation of injectable osteogenic tissue constructs is another critical issue in bone stem cell therapy. Regarding this, Zhao et al. encapsulated bone-marrow-derived mesenchymal stem cells (BMSCs) and bone morphogenetic protein-2 (BMP-2, as an osteogenic growth factor) inside photo-crosslinkable gelatin methacryloyl (GelMA) microspheres with a high-speed production rate using a capillary microfluidic device. Incorporating BMP-2 into GelMA microgels improved stem cell proliferation, osteogenesis, and mineralization in both in vitro and in vivo studies. These cell-laden structures supported the spreading of stem cells within the microspheres and their migration out of the microspheres [98]. In another study, Hou et al. prepared injectable, degradable poly(vinyl alcohol) (PVA) microgels (100–200 µm in diameter) loaded with hMSCs and BPM-2 via high-throughput microfluidics-based systems. The joining of this growth factor to PVA microgels increased ALP activity, calcium content, and Runx2 expression in encapsulated cells. The mild crosslinking and cell-compatible conditions enabled the encapsulation of hMSCs with prolonged survival, differentiation, and migration [99]. Other studies encapsulated single stem cells inside microspheres for the purpose of osteogenic differentiation. For example, An et al. encapsulated rat MSCs at a single-cell level inside alginate microspheres. The immune-shielding properties of he alginate shell supported bone formation after transplantation to the rat tibial ablation model (Figure 5A) [100].

### 4.2. Cartilage Tissue Engineering

The direct injection of stem cells with hydrogels to the damaged articular cartilage is considered a potential therapeutic method [101]. The hydrogels prepared based on bulk fabrication methods face encapsulated cells with significant challenges, including high apoptosis rate, low retention in the host cartilage, and inadequate chondrogenic differentiation and functionality. A variety of microfabrication methods have been used to encapsulate cells for cartilage regeneration, among which a limited number are based on droplets. For instance, Li et al. encapsulated BMSCs inside visible light-cured microgels composed of gelatin norbornene (GelNB) and polyethylene glycol (PEG), and differentiated them into chondrocytes in chondroinductive media. The encapsulation process using droplets increased the chondrogenesis of cells compared to the bulk hydrogel and 2D culture [102]. In an in vivo study, Feng et al. designed and fabricated injectable cell-laden microgels to self-assemble in situ inside the injury point and from a 3D porous scaffold without external stimulation; they encapsulated BMSCs inside these hybrid microspheres composed of gelatin and hyaluronic acid (HA) microgels via a thiol-Michael addition reaction. These cell-laden microgels self-assembled into cartilage-like structures via cell–cell interconnectivity, and supported proliferation and chondrogenic differentiation of encapsulated BMSCs. Furthermore, the occurrence of vascularization and hypertrophy—which are both considered huge barriers in cartilage regeneration—decreased significantly after transplantation (Figure 5B) [103].

In addition to the cell-encapsulating role of droplets, these microstructures can be employed for the sustained release of biological cues inside the injury point. For instance, the transforming growth factor beta-3 (TGF-β3)—a cytokine—plays an essential role in increasing the viability and ECM secretion of chondrocytes, and in enhancing the chondrogenic differentiation of stem cells. In this regard, Qasim et al. loaded poly(L-lactic-co-glycolic acid) (PLGA) particles with TGF-β3 using a droplet-based microfluidic system, and incorporated them into polycaprolactone (PCL) nanofibers using the electrospinning method. The fabricated microspheres improved the loading efficiency of TGF-β3 by up to 80%. They found that these microparticles blended with nanofibrous scaffolds promoted the proliferation and chondrogenic differentiation of hMSCs [104].

### 4.3. Wound Healing

As the largest organ in terms of surface area in the body, skin protects internal tissues from various damaging elements, including mechanical injuries, infections, ultraviolet radiation, and temperature. Severe skin-related injuries endanger the patient’s life and negatively impact the healthcare economy [105]. Even though various therapeutic methods for wound healing have been developed in the clinic over the past decade, these approaches are practical only for moderate injuries. The arrival of advanced skin tissue engineering methods helps to regenerate damaged parts [106]. For this aim, researchers developed scaffolds using droplet-based microfabrication techniques for severe skin damage. For instance, Yu et al. fabricated hollow microspheres of bacterial cellulose using a droplet-based fabrication process, and applied them as wound-healing materials for the regeneration of significantly damaged skin. To this end, they encapsulated *Gluconacetobacter xylinus* bacteria in core–shell microspheres with an alginate core and an agarose shell. The bacteria inside the core secrete and tangles cellulose in the shell part. The alginate core and bacteria are removed, and hollow cellulose microspheres remain in the structure. These porous microspheres showed acceptable mechanical properties that supported the proliferation and migration of PC-9 cells. Furthermore, in collaboration with human primary epidermal keratinocytes, these scaffolds enhanced wound healing in male Sprague Dawley rats [107].

The fabrication of hydrogel microparticles permits direct delivery of cells through needles to the injured tissue for wound healing with the least clinical invasiveness. In this regard, Griffin et al. synthesized a new class of injectable microporous annealed-particle gels that enabled a stable, interconnected network of micropores for cell migration. They prepared complex three-dimensional networks based on self-assembly of monodisperse microgel building blocks made using a microfluidic device (Figure 5C). The annealing of these building blocks created an interconnected microporous scaffold, in which cells could be placed in the interconnected pores. Furthermore, the building blocks were easily injected into the mice’s bodies, and caused faster skin regeneration and lower immune response than non-porous scaffolds after one week. The nature of these droplet-based building blocks allows the combination of an extensive range of materials, signals, and cell populations [108].

### 4.4. Pancreas Regeneration

Diabetes mellitus is one of the most prevalent metabolic diseases all over the world. The in vitro culture and transplantation of pancreatic islets is considered an effective therapeutic method to aid patients with type I diabetes. Droplet-based encapsulation methods can increase the safety of the encapsulation process, control the size of scaffolds, permit long-term culture periods, and prepare immunological protection of these pancreatic islets in the engineering of pancreatic tissue [109].

Fabrication of core–shell scaffold structures using microfluidics-derived droplets can provide islet cells with more immunoprotection after injection into the host. For instance, Ma et al. encapsulated rat pancreatic islets with high viability in alginate core–shell microcapsules and injected them into the type I diabetic mouse model. The transplantation of cell-laden core–shell structures decreased blood glucose to the normal range without stimulating the immune system [110]. In another study, Headen et al. encapsulated human pancreatic islets in scaffolds made of maleimide-functionalized PEG (PEG-4MAL) via a flow-focusing microfluidic system. The encapsulated cells inside scaffolds showed viability of 90% after eight days of culture (Figure 5D). On the other hand, capsules showed a selective permeability to biomolecules such as glucose, insulin, and antibodies [111]. The presence of oils and surfactants in droplet fabrication procedures can endanger the viability and functionality of encapsulated cells. In this regard, Lue et al. encapsulated rat pancreatic islets (β-TC6) inside calcium alginate hydrogels using a water-in-water system; they used a pneumatic valve combined with a crossflow microfluidic device to assist in the production of droplets and improve controllability. The encapsulated cells responded to the stimulation of glucose during cultivation for seven days [112].

### 4.5. Liver Regeneration

More than half a billion people are suffering from various liver diseases worldwide [113]. Liver transplantation is still the only therapeutic method for severe hepatic disorders, and patients face a severe shortage of donors and a long waiting list. Thus, regenerative-medicine-based methods are in high demand in the field of liver regeneration [114]. The morphology of hepatic cells, their cell–cell and cell–scaffold interactions, and the capability of spheroid formation, albumin secretion, and urea synthesis are the main challenges in liver regeneration, and are highly dependent on the microenvironment in which the cells are encapsulated.

The hepatic spheroid-laden hydrogels are of great interest in liver regeneration. One of the greatest limitations of the conventional microencapsulation method is that a constant number of spheroids cannot be encapsulated and produced per microgel. Chan et al. encapsulated hepatocyte spheroids inside alginate microgels using a droplet-based system. The gelation time of hydrogel permitted hepatocytes to improve their assembly within the liquid core. Additionally, their study showed that the co-culturing of hepatocytes with endothelial progenitor cells (5:1) improved the hepatic functionality, differentiation, angiogenesis, and long-term performance of cells (Figure 5E) [115].

The cell-laden spheres simulate the niche of hepatic cells, and also protect them against the immune system. In this regard, Wang et al. fabricated core–shell cell-laden microgels made of methylcellulose and GelMA. The device incorporated HepG2 cells with and without HUVECs through a methylcellulose core. This core was encapsulated in a GelMA shell, and microgels were cultured for a two-week in vitro study. The presence of HUVECs enhanced the functionality of hepatocytes, which can be related to homotypic and heterotypic cell–cell interactions in such microenvironments [116].

### 4.6. Cardiovascular Regeneration

Heart engineering using various scaffolds has been investigated as an alternative for managing cardiovascular diseases in the future [117]. Droplet-based encapsulation of cells can provide controllable biocompatible microenvironments to fabricate scaffolds with desirable mechanical properties for cardiovascular tissue engineering. For instance, Gal et al. encapsulated cardiac cells in personalized spherical hydrogels made of human acellular omentum and transplanted them to murine muscle. After transplantation, the encapsulated cells were spread at the injection site, exhibiting striation of actinin and connexin-43 (Figure 5F) [118]. Droplets can also serve as a temporary shield for preserving cells during severe processes such as electrospinning. Weidenbacher et al. fabricated droplets to temporarily protect murine myoblast cells via their encapsulation in gelatin microspheres for the electrospinning process. These microcapsules were electrosprayed onto the surface of nanofibrous polyvinylidene fluoride-co-hexafluoropropylene sheets to make a hybrid scaffold. The encapsulated cells were protected from the toxicity of dimethylformamide during electrospinning [119].

A variety of native biomolecules can be used to enhance cellular attachments. For instance, RGD is an integrin-binding peptide found as an adhesion motif in many ECM proteins—including fibronectin, fibrinogen, and von Willebrand factor (VWF)—commonly used to increase cellular regeneration [120]. Therefore, controlled functionalization of cell-encapsulated microparticles with adhesive peptides using cytocompatible crosslinking agents affords an environment capable of long-term cell viability. Such a microenvironment may be practical for either cell encapsulation or directing stem cell behavior and fate. In another study by Cha et al., a microfluidic flow-focusing device was utilized to fabricate GelMA microgels by photo-crosslinking UV light as a highly promising injectable tissue construct. The variable ratios of flow rates of aqueous and oil phases could control the droplet size. Then, the cardiac population cells were cultured on the GelMA microgel’s surface. The adhered cells on the microgel surface proliferated over time, while maintaining high viability (∼90%) and migrating to their cell-conductive surrounding areas. Furthermore, a thin biocompatible and biodegradable silica hydrogel was coated on the cell-seeded microgels’ surface via the sol−gel method, as a protective shell against peroxide-induced oxidative stress during and after implantation in host tissues. The silica hydrogel shell degraded over time, without affecting cellular activities [121].

The endothelial tissue plays a crucial role in regulating homeostasis in the cardiovascular system. The monolayer of endothelial cells is a kind of biological barrier between blood and tissues; this tissue regulates different immunohematology processes in the body, including secretion of vasomotor and growth factors, and starting mechanisms of inflammation and clotting in vascular injuries [122]. This semi-permeable barrier dynamically regulates the transportation of different elements between blood and the underlying tissue. For measuring endothelial cells’ functions in a microenvironment close to in vivo conditions, Crampton et al. generated collagen microparticles using a microfluidics-based droplet system and coated the surfaces with endothelial cells. They found that endothelial cells on the surface of microspheres showed typical morphology and produced tight junction proteins; their results also revealed that these cell layers had a permeability similar to that observed in vivo, and were responsive to modulators of endothelial permeability such as tumor necrosis factor-alpha (TNF-α) and TGF-β [123].

## 5. Microfluidics-Based Droplets for Assisted Reproductive Technology

Infertility is an ever-increasing public health concern worldwide, leading to the concern that more than 70 million couples worldwide have experienced infertility issues at least once in their life [124]. A variety of therapeutic methods have been developed to assist patients in the clinic. For instance, the development of mammalian reproductive technologies—most notably ART—has been one of the most outstanding achievements in the treatment of infertility [125]. ART and its different subcategories—including ovulation induction, intrauterine insemination (IUI), and in vitro fertilization (IVF)—provide an extraordinary chance for infertile couples to have offspring. Combining ART with stem cell therapy and tissue engineering approaches—such as scaffold fabrication techniques, cell encapsulation, 3D culture microenvironments, and microfluidics—could lead to the development of artificial reproductive organs, stem-cell-derived gametes, or even human cloning [125,126,127].

Microfluidic platforms based on single-phase continuous flow have been successfully used in various ART fields, including infertility diagnosis, sperm selection, sperm guidance, oocyte analysis, insemination, embryo culture, embryo selection, and cryopreservation. Recent research interest has been shifted toward microfluidics-based droplets, since they can offer well-controlled environments for the encapsulation of cells and other elements necessary for ART operations inside micro-scale droplets [128]. The fabrication and handling techniques of droplet-based ART systems can be divided into emulsion-based droplet microfluidics and digital microfluidics.

In emulsion-based droplet microfluidics, the droplet can automatically be formed by the interaction of two immiscible fluids, automatically generating individual micro-bioreactors from the cell suspension. For ART applications, this microfluidics category is very similar to what embryologists perform in clinical IVF, such as encapsulating the gamete in a microdroplet covered with an oil overlay. These droplets can also act as platforms for culturing embryos in the early stages. For instance, Agarwal et al. devised a core–shell encapsulation technique for 3D culture of embryonic cells) [70]. Moreover, this technique has been implemented for in vitro culturing of ESCs and ovarian preantral follicles with alginate and type I collagen as shell and core structures, respectively [129]. Moreover, the micro-segmented flow systems can be used for gamete encapsulation and culture. The most relevant work in this field was reported by Funfak et al., who used the microfluid segment technique to encapsulate the eggs of zebrafish until hatching time in a polytetrafluoroethylene (PTFE) tube, with perfluoromethyldecalin (PP9) as the carrier liquid [130].

Digital microfluidics is another promising field for precisely generating and manipulating droplets by implementing noncontact forces such as electricity, magnetism, or heat for ART and IVF applications, such as gamete manipulation and embryo culture. In this regard, two standard digital microfluidics techniques have been used in the literature: electrowetting on dielectric (EWOD), and liquid marbles (LMs). EWOD is a facile method to manipulate discretized droplets on a solid surface in a programmable manner, using electrodes made of a conductor such as indium tin oxide (ITO). In a recent study, murine embryos were encapsulated in a culture medium (3.5 µL) as a core and an oil droplet (1.5 µL) as a shell at the bottom of an EWOD plate. The EWOD technique can also be used for slow-freezing cryopreservation (SFC) and vitrification of cells, tissues, and embryos. For instance, Park et al. showed that EWOD digital microfluidics could be successfully used to select the optimal mixture of cryoprotective agents for efficient SFC [131].

Liquid marbles can also be regarded as another emerging digital microfluidics technique for the straightforward and facile manipulation of water droplets on solid surfaces [132]. A liquid marble can be directly prepared by rolling water droplets on hydrophobic materials such as PTFE or lycopodium and manipulating them with various external forces [133]. Furthermore, they can be used as bioreactors for 3D spheroid formation. For instance, Sarvi et al. demonstrated the feasibility of forming uniform EBs from Oct4B2-ESCs within a three-day culture inside liquid marbles; they also showed that these microenvironments could facilitate the differentiation of embryo bodies into cardiomyocytes. Furthermore, these droplets can be implemented as an efficient micro-bioreactor for in vitro oocyte maturation in ART [134].

## 6. Microfluidics-Based Droplets for Gene Therapy/Delivery

Gene therapy/delivery is considered one of the most promising methods in advanced regenerative medicine [135]. Mutated or missing genes result in the expression of dysfunctional or abnormal intra- and extracellular proteins that eventually lead to many diseases. As a valuable method, gene therapy was developed to correct genetic disorders by inserting genetic materials into cells. The exogenous genetic materials must be delivered across the cell membrane without any influences on the ability of cells to integrate successfully into the innate cell genome [136]. Regarding the continuous lipid bilayer structure of the spherical cell membrane, the transfer of foreign genetic materials through the plasma membrane is a stumbling block in gene therapy. Several physical techniques—such as sonoporation and electroporation—create temporary pores in the cell membrane using ultrasound and electric pulses. Although electroporation is the most popular method of intracellular delivery, the efficiency of sonoporation systems increases when attached to microbubbles. Concerning the downsides of conventional gene transfection systems—such as low transfection efficiency, intricate procedure, and low cell viability—the advent of microfluidics has opened up new avenues for gene delivery [21].

Gene delivery through microfluidic systems is subject to high precision and outstanding control that can finally cause the efficient generation of various vectors and other components used in gene therapy. Several genes in bulk solution have been explored by implementing a microfluidic environment combined conventionally with an electric pulse, optical energy, and hydrodynamic force. Moreover, most microfluidics-based electroporation systems require much lower voltages for gene transport, resulting in higher efficiency and cell viability than traditional electroporation methods [137]. Several microfluidic geometries, diverse materials, electrodes, and microfluidic channels have been reported to increase the insertion of gene materials into cells via electroporation. However, further development for increased efficiency is required.

**Figure 6:**
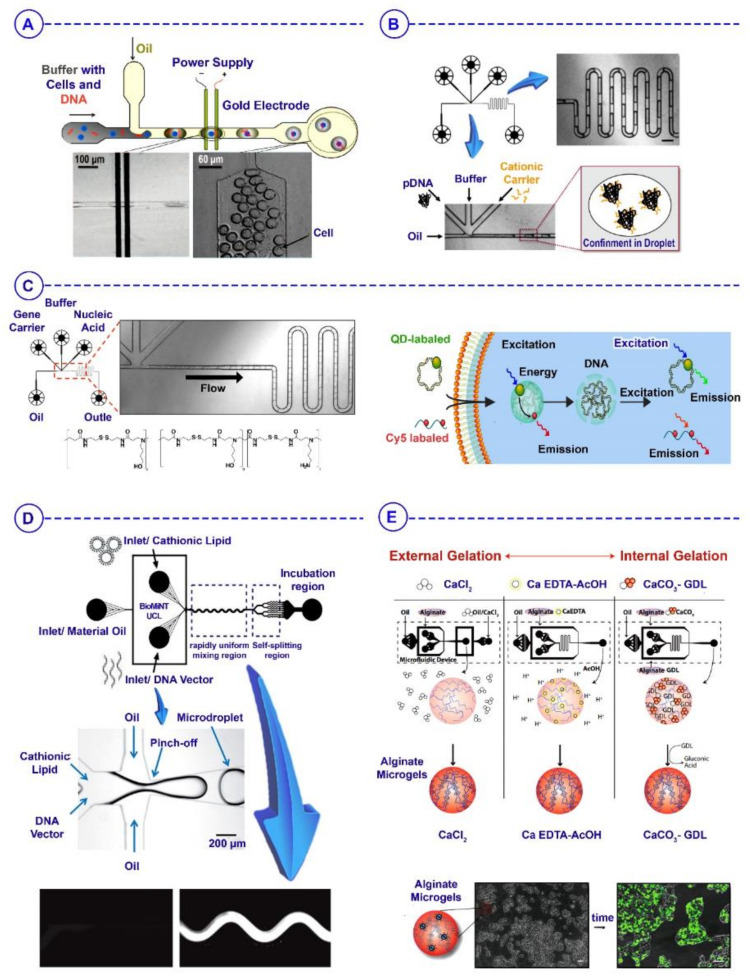
Microfluidics-based droplets for gene therapy/delivery: (**A**) Design of a microfluidic device for electroporation. Cell- and DNA-containing droplets rapidly flow through the two microelectrodes on the substrate and droplets with encapsulated cells after electroporation at the device’s exit [139]; Copyright 2009, Wiley Online Library. (**B**) Design of microfluidics-assisted self-assembly for fabricating picoliter-sized droplets. The plasmid DNA, buffer, cationic gene carrier, and oil are introduced into each channel to generate monodisperse water-in-oil droplets (scale bar: 200 μm) [140]; Copyright, American Chemical Society. (**C**) Design of a microfluidic chip for non-viral transport of genes, as well as the cellular internalization and intracellular unpacking of genes. The microfluidic system uses crossflow geometry to produce emulsified aqueous droplets containing the polymeric gene carrier and nucleic acids [141]; Copyright, Nature. (**D**) Design of the picoliter microfluidic reactor and incubator system for controlled formulation of non-viral vectors (CL-DNA) [143]; Copyright 2009, The Royal Society of Chemistry. (**E**) Representative phase-contrast photomicrographs of alginate microgels fabricated using three different gelation techniques [144]; Copyright, The Royal Society of Chemistry.

Recently, new methods with a combination of microfluidics-derived droplets and conventional gene transfection techniques have emerged, with the ability to enhance the accuracy of transportation of exogenous genetic factors at a single-cell level [138]. For instance, using these methods, Zhan et al. delivered an enhanced green fluorescent protein (EGFP) plasmid vector into Chinese hamster ovary (CHO) cells; they encapsulated CHO and EGFP plasmids inside droplets, which then passed through a pair of microelectrodes with a constant voltage. The electroporated cells’ GFP expression proved high-throughput insertion of functional genetic materials based on droplet microfluidics (Figure 6A) [139]. In another study, nanocomplexes were synthesized using microfluidics-assisted confinement (MAC) for non-viral vector delivery of genes. A complicated microfluidics-based droplet generator was applied to introduce various materials—such as cationic gene carriers, plasmid DNA, buffers, and oils—into each channel to generate monodisperse droplets. To form DNA nanocomplexes, the pDNA and polycation solutions were confined to discrete droplets and successfully self-assembled electrostatically (Figure 6B) [140]. Upon incubation with human embryonic kidney cells (HEK293), these homogeneous MAC-generated nanocomplexes exhibited lower cytotoxicity and higher transfection efficiency than their bulk-synthesized counterparts. To further explore the merits of the MAC system for non-viral gene transfer, another experiment in 2013 revealed that the emulsion-based droplets’ microfluidic environment offers greater control of the preparation of polyplexes to generate more constant potential gene delivery systems (Figure 6C) [141]. By operating such a system, both plasmid DNA and messenger RNA payloads are consistently delivered into primary cells, stem cells, and human cell lines. The cellular unpacking of polymer–DNA nanocomplexes quantified by a flow cytometric quantum dot Förster resonance energy transfer (QD-FRET) nanosensor system demonstrated that transfection was significantly enhanced in a broad series of cell types in terms of both uncultured transgene expression and the number of cells transfected. Yang et al. also demonstrated that polymer-based nanoparticles using the dielectrophoresis (DEP) effect could be generated inside droplets within a roughly nanoliter-scale volume. They used a sort of nanomaterial for tumor-targeted gene therapy—PEI600-CyD-FA (H1)—and then mixed it with DNA plasmids to produce polyplex products that can be self-assembled. These nano-scale polymer-based particles were examined in HUVECs, and the results demonstrated that the gene transfection efficiency increased fourfold compared to the control group [142].

The picoliter microfluidic reactor and incubator (PMRI) system is the other droplet-based gene transfection method that can control the cationic lipid and DNA complex (CL-DNA) by tuning of the mixture incubation time and the order and rate of mixing of vectors. Hsieh et al. used human osteosarcoma U2OS cells (ATCC) with the pEGFP-C1 DNA vector to characterize the transfection efficiency of the prepared CL-DNA via the PMRI technique (Figure 6D); their results demonstrate that PMRI consistently mixes cationic lipids and DNA simultaneously to create a narrower lipoplex scattering. The latter, however, makes it essential to recognize the optimal lipoplex size for extreme transfection efficiency [143]. Droplet-based microfluidics may also provide a platform for viral vector delivery. In a study reported by Madrigal et al., alginate and LentiVector were incorporated into microfluidic technology to create LentiVector-loaded microgels using different gelation methods. Comparisons of three alginate gelation strategies revealed that internal gelation with CaCO_3_/D-glucono-δ-lactone (GDL) and external gelation with CaCl_2_ are suitable for creating LentiVector-compatible microgels. In contrast, alginate gelation with chelated calcium confirmed low utility for gene delivery due to a loss of LentiVector function under acidic gelation conditions (Figure 6E). These results demonstrate alginate microgels’ capacity to successfully encapsulate and release functional LentiVector for gene delivery in vitro [144].

In conclusion, microfluidics-assisted gene transfection systems have achieved higher transfection efficiency and cell viability than conventional gene transfection processes. The microfluidics-based droplet environment allows for better spatiotemporal control over the environment. The strength and duration of the transfection stimulus eliminate the randomness of traditional transfection methods in the target cells. A further improvement would cause applied droplet-based microfluidics technology for long-term single-cell culturing, homogenous transfection, and single-cell sorting to replace the complicated operation procedures involved in gene transfection in conventional benchtop systems.

## 7. Conclusions and Perspectives

The translation of regenerative medicine to clinical practice is highly dependent on advanced technologies, manufacturing methods, clinical trial success, and commercial support. Microfluidics-based droplets are a promising technology that enables the simultaneous encapsulation of various stem cells, biological agents, drugs, and nanoparticles inside specifically designed droplets for advancing integrated aspects of regenerative medicine. This review article evaluated the state-of-the-art microfluidics-based strategies for encapsulating stem cells inside droplets for stem cell therapy, tissue engineering, reproductive biology, and gene therapy (Table 1).

In droplet-based stem cell therapy, preserving the viability of stem cells during the manufacturing process is a challenge that can be addressed by implementing nontoxic biomaterials, increasing automation, and reducing manufacturing time. The challenges in stem-cell-based therapy—such as the risk of tumorigenicity and immunogenicity—have not been widely investigated enough (during in vivo and clinical trial studies) for droplets carrying stem cells, spheroids, and organoids. Furthermore, there are no established standards or procedures for droplet-based stem cell therapy. Recent studies show that the regeneration caused by stem cells is mainly caused by secretome proteins, exosomes, and EVs generated by transplanted stem cells [145]. This knowledge can be implemented to generate secretome-enriched droplets or droplet-derived microgels for cell-free regenerative medicine, thus reducing the possible side effects caused by the transplantation of stem cells.

The human body’s tissues, organs, and systems are developed based on a complicated innate bottom-up organization [146]. It is essential to mimic the target tissue’s conditions and build repair-tissue-like building blocks in the same manner in order to regenerate the tissues. Despite the several reports and advances in this area, the scaffolds and microgels made by droplets still cannot mimic many aspects of tissues, such as transport of fluids, transport of electrophysiological signals, angiogenesis, and neovascularization. The available polymer options are limited to some hydrogels for safely encapsulating stem cells via this approach, and need to be expanded and evaluated in future studies [76]. Moreover, scaffolds’ mechanical strength plays an essential role in tissue engineering, while the natural hydrogels used in reported microgels have weak mechanical properties that are not desirable for hard tissues [92]. The possible adverse effects of crosslinkers, oils, and mechanical forces during the encapsulation process and injection should be investigated during long-term in vitro and in vivo studies. In addition to the fact that the retention of the cells in the tissue is a critical challenge, the retention of cell-laden microgels and their integration and unification with the damaged tissue after injection is also essential, and can be addressed using surface modification methods of microgels for self-assembly purposes [140]. The number of in vitro and in vivo studies implementing droplet-derived microgels for tissue engineering is considerably fewer than those using conventional scaffolds. In reproductive biology, the automation of droplet-based systems and improvement of digital microfluidic systems with programmable hardware is essential for removing the human errors during complicated droplet formation and manipulation procedures, gamete isolation, manipulation, culture, fertilization, and embryo culture [131]. The high sensitivity, accuracy, and control in delivering specific genes to the individual target cells inside droplets using advanced operations and instruments are still highly expensive, and the cost needs to be reduced for commercialization purposes. The studies related to these fields should also be expanded to in vivo and clinical trials.

Here, the applications of microfluidics-based droplets in the fields of stem cell therapies, tissue engineering, reproductive biology, and gene therapy have been reviewed and discussed. Despite the considerable achievements in droplet-based regenerative medicine, the field is expected to develop significantly in the future, removing the gaps between lab and clinic in each area. In the first step, for exploiting these droplets or their products in clinical procedures, significant issues should be considered, including automation and medical costs, preparing hygienic conditions, clean rooms, advanced manufacturing instruments, and Food and Drug Administration (FDA)-approved materials, and following regulatory necessities and ethical issues. Soon, droplets will be able to revive the dream of preparing off-the-shelf products for curing patients with mild or severe dysfunctionalities.

## Figures and Tables

**Table 1:** Applications of droplet-based microfluidics for stem cell therapy, tissue engineering, assisted reproductive biology, and gene therapy.

Field	Application	Generation Technique	Droplet Size	Morphology/ Materials	Cell Type	Ref
Stem Cell Therapy	Stem cell culture	Flow-focusing; two-layer parallel system	64–65 μm	Microspheres	MSCs	[57]
		Air-driven droplet generator	400–500 μm	Alginate microcapsules	hESCs	[63]
	Spheroid culture	Flow-focusing	65–90 μm 150–210 μm	Alginate and alginate/RGD	hMSCs; spheroids	[69]
		Flow-focusing	190–260 μm	Alginate microcapsules	Ambryonic bodies	[70]
		Flow-focusing: - Negative pressure-driven - Micropipette-based	290 µm	Droplets	HEK293; RT4 cells; A-431 cells	[71]
		Flow-focusing	-	Droplets	Primary rat hepatocytes	[72]
	Organoid culture	Flow-focusing	169 ± 6 μm	Alginate capsules	Hepatocytes fibroblasts	[73]
		Airflow-assisted 3D bioprinting; flow-focusing	-	Spiral alginate: - Spherical - Rose-like - Tai-chi-like	HUVECs; hMSCs	[74]
	High-throughput analysis	Flow-focusing	-	Droplets for RNA sequencing	ESCs	[85]
		Flow-focusing	10–150 μm	Droplets for ELISA	Hybridoma cells	[87]
Tissue Engineering	Bone	Flow-focusing	427 µm	RGD; alginate	PDLSCs; GMSCs	[97]
		Capillary-based	90–230 µm	BMP-2; GelMA	BMSCs	[98]
		Flow-focusing	100–200 µm	BMP-2; PVA	hMSCs	[99]
		Flow-focusing	61.2–50 µm	Alginate	Single MSCs	[100]
	Cartilage	Capillary-based	320 ± 9 µm 574 ± 9 µm	Gelatine norbornene; PEG	BMSCs	[102]
		T-junction with Y-shaped inlets	-	Gelatine; hyaluronic acid	BMSCs	[103]
		Flow-focusing	10–30 µm	TGF-β3/PLGA droplets for PCL fibres	hMSCs	[104]
	Wound	Flow-focusing	20, 40, 50 µm	Alginate core, cellulose shell	Gluconacetobacter xylinus PC-9 cells	[107]
		Flow-focusing	30–150 µm	4-arm PEG vinyl	HDF; hMSCs	[108]
	Pancreas	Coaxial electrojetting	500 µm	Core–shell alginate	Pancreatic islets	[110]
		Flow-focusing	300–800 μm	PEG-4MAL	Human pancreatic islets	[111]
		Eight flow-focusing orifices	90.4 ± 3.0 µm	Alginate	β-TC6	[112]
	Liver	Flow-focusing	≈200 µm	Alginate	Hepatocytes; endothelial cells	[115]
		Flow-focusing	289.7 ± 8.3 µm	Methylcellulose GelMA	HepG2; HUVECs	[116]
	Cardiac /vascular	Flow-focusing	100–200 µm	Acellular omentum	iPSCs-derived cardiac cells	[118]
		Flow-focusing	40–140 µm	Gelatine	Murine myoblast cells	[119]
		Flow-focusing	35–150 µm	GelMA; silica hydrogel	Cardiac cells	[121]
		Flow-focusing	300 µm	Collagen	Endothelial cells	[123]
Assisted Reproductive Technology		Flow-focusing	380 µm 285 µm	Core–shell collagen alginate microcapsules	Ovarian preantral follicles; ESCs	[129]
		-	1.2 mm	PP9	Zebrafish eggs	[130]
		Electrowetting on dielectric (EWOD)	-	f DMSO-PBS mixture	MCF-7	[134]
Gene Therapy/ Delivery	Electroporation	T-junction	92 µm	EGFP plasmid vector droplets	Chinese hamster ovary cells	[139]
	Microfluidics-assisted confinement	Crossflow	0.5–1 μm	Plasmid DNA droplets	-	[140]
	Gene transfer	Crossflow	-	Plasmid DNA and messenger RNA droplets	Primary cells; stem cells; human cell lines	[141]
	Gene delivery	T-junction	-	PEI600-CyD-FA (H1) DNA plasmids; polyplexes; droplets	HUVECs	[142]
	Gene transfection	Flow-focusing	39 μm	pEGFP-C1 DNA vector	Human U2OS cells	[143]
	Viral gene transfection	Flow-focusing	100–130 μm	Alginate; gelation; LentiVector	HEK-293T	[144]
	CRISPR/Cas9 transfection	Flow-focusing	80–87 µm		hiPSCs	[138]
